# Coenrollment of critically ill patients in PROSPECT: characteristics and association with treatment efficacy and safety

**DOI:** 10.1186/s13063-025-09028-w

**Published:** 2025-09-26

**Authors:** Alex Thabane, Diane Heels-Ansdell, Nicole Zytaruk, Jennie Johnstone, François Lauzier, Yaseen M. Arabi, John Muscedere, France Clarke, Lori Hand, Irene Watpool, Rebecca Porteous, Gyan Sandu, Marlene Santos, Danae Tassy, John Marshall, Lauralyn McIntyre, Ian Ball, Bram Rochwerg, Tim Karachi, Ryan Zarychanski, Francois Marquis, Jan O. Friedrich, Paul Lysecki, Deborah Cook, For the PROSPECT Investigators and the Canadian Critical Care Trials Group, Nicole Zytaruk, France Clarke, Lori Hand, Lois Saunders, Shelley Anderson-White, Alyson Takaoka, Mary Copland, Megan Davis, Neala Hoad, Melissa Shears, Kristine Wachmann

**Affiliations:** 1 Department of Health Research Methods, Evidence, and Impact, McMaster University, Hamilton, Canada; 2 Department of Medicine, University of Toronto, Toronto, Canada; 3 Department of Medicine, Université Laval, Québec City, Canada; 4 King Saud Bin Abdulaziz University for Health Sciences and King Abdullah International Medical Research Center, Riyadh, Saudi Arabia; 5 Department of Critical Care Medicine, Queen’s University, Kingston, Canada; 6 Department of Critical Care Medicine, University of Ottawa, Ottawa, Canada; 7 Interdepartmental Division of Critical Care Medicine, University of Toronto, Toronto, Canada; 8 Hôpital Maisonneuve-Rosemont, Montréal, Canada; 9 Department of Critical Care Medicine, Western University, London, Canada; 10 Department of Medicine, McMaster University Medical Center, 1280 Main St W, Hamilton, ON L8S 4L8 Canada; 11 Department of Critical Care Medicine, University of Manitoba, Winnipeg, Canada; 12 Department of Critical Care Medicine, Joseph Brant Hospital, Burlington, Canada

**Keywords:** Probiotics, Coenrollment, Randomized trial, PROSPECT, Critical care

## Abstract

**Introduction:**

Coenrollment is the enrollment of one participant into more than one study. While coenrollment can enhance research efficiency, it theoretically may result in treatment interactions that distort effect estimates. This study aimed to explore the sensitivity of safety and efficacy outcomes to coenrollment in an international, blinded randomized controlled trial evaluating probiotic use in critically ill patients (PROSPECT: Probiotics: Prevention of Severe Pneumonia and Endotracheal Colonization Trial; [NCT02462590]).

**Methods:**

In this planned secondary analysis of PROSPECT, we performed Cox proportional hazards analyses to assess the sensitivity of the treatment effect of probiotics to coenrollment on the primary outcome of ventilator-associated pneumonia. Secondarily, we examined the characteristics of coenrolled patients, studies, and centers using descriptive statistics, explored factors associated with coenrollment via a multilevel logistic regression model, and conducted Fisher’s exact tests to evaluate the difference in adverse event rates (defined as *Lactobacillus* species from a sterile site or cultured as the sole or predominant organism from a nonsterile site) by coenrollment status.

**Results:**

Of 2650 PROSPECT participants recruited across 44 centers, 568 patients (21.4%) were coenrolled a total of 680 times across 115 studies. Coenrollment did not modify the effect of probiotics on the primary outcome of ventilator-associated pneumonia (*p* = 0.630). Patients who were coenrolled in any other study had a higher rate of adverse events compared to non-coenrolled patients (*p* = 0.011); however, post hoc testing found no difference in adverse events between patients coenrolled specifically into at least one other randomized controlled trial and patients who were not coenrolled into another randomized controlled trial (i.e., coenrolled into an observational study or not coenrolled at all; *p* = 0.126). Multivariable analyses found more severely ill patients (*p* = 0.038) and patients from centers with a longer PROSPECT recruitment period (*p* = 0.047) were more likely to be coenrolled.

**Conclusion:**

In this international, blinded trial, one-fifth of patients enrolled were coenrolled in at least one other study, which had no influence on the effect of probiotics on the primary outcome. Coenrolled patients were more likely to have higher disease severity, and to be recruited from a center with a longer history of participation in PROSPECT.

**Trial registration:**

The PROSPECT trial was registered in ClinicalTrials.gov NCT02462590. Registered on June 2015.

**Supplementary Information:**

The online version contains supplementary material available at 10.1186/s13063-025-09028-w.

## Background

Systematic reviews and randomized trials have found probiotics to be efficacious in the prevention or treatment of many conditions including diarrhea [1–3], gastrointestinal diseases [4], respiratory tract infections [5–7], allergic rhinitis [8], dermatitis [9, 10], and eczema [11]. Early evidence of the clinical utility of probiotics was also reported in the field of critical care for the prevention of ventilator-associated pneumonia (VAP) [12], a serious infectious complication associated with a high risk of morbidity and mortality [13]. However, the recent PROSPECT trial (Probiotics: Prevention of Severe Pneumonia and Endotracheal Colonization Trial), which randomly allocated 2650 invasively mechanically ventilated critically ill patients to receive either the probiotic *Lactobacillus rhamnosus* GG or a placebo, showed no effect on VAP [14].

While subsequent systematic reviews of randomized trials showed a reduction in VAP with probiotics, sensitivity analyses excluding low-quality studies found no benefit of probiotics in reducing VAP [15, 16]. The inconsistent results between PROSPECT and other individual trials, as well as analyses reported in recent systematic reviews, suggest the need to further explore the robustness of the PROSPECT findings. One possible effect modifier is *coenrollment*, defined as the simultaneous or sequential enrollment of one patient into more than one study.

There is ongoing discussion among trialists on the risks and benefits of coenrollment in clinical research. Coenrollment offers certain research advantages, including a lower risk of selection bias into trials and improved efficiency [17, 18]. However, coenrollment may cause unintended interactions between trial interventions, thereby modifying the observed treatment effects in the involved trials [18]. The few studies of coenrollment in critical care have found minimal effect on trial outcomes [18–20]. While this suggests that coenrollment may not influence trial outcomes, continued scientific investigation of the effects of coenrollment on trial processes and outcomes is required, especially given the highly vulnerable nature of critically ill patients. The large, international PROSPECT trial collected data on patient coenrollment and represents an opportunity to examine these issues, whilst also exploring the robustness of the trial results which showed no benefit of probiotics in reducing VAP, in contrast to prior trials evaluating probiotics in critically ill patients.

The primary objective of this study was to evaluate the interaction between treatment effect and coenrollment status on the primary outcome of VAP among patients enrolled in the PROSPECT trial. Secondary objectives were focused on factors associated with coenrollment and consequences of coenrollment.

## Methods

### Study design

This study is a planned secondary analysis of the PROSPECT randomized controlled trial [14]. The feasibility pilot trial design and results, as well as the statistical analysis plan and results for the main PROSPECT trial, have been published [14, 21–23]. The current study examining coenrollment is guided by its own protocol, pre-specified hypotheses, and detailed statistical analysis plan (Supplementary Table 1) [24].

### Primary objective

The primary objective of this study was to evaluate the effect of patient coenrollment on the treatment effect of probiotics on VAP in invasively ventilated patients.

### Secondary objectives

Our secondary objectives were to describe the characteristics of coenrolled patients and coenrolled studies; explore differences in baseline characteristics between coenrolled and non-coenrolled patients; explore differences in center-level characteristics between coenrolling and non-coenrolling centers; identify factors associated with coenrollment; and explore the relationship between coenrollment status and the incidence of adverse events.

### Summary of the PROSPECT trial

Patients were enrolled in a total of 44 intensive care units (ICUs) in Canada (41 ICUs), the United States (2 ICUs), and Saudi Arabia (1 ICU) [14, 22]. Patients were adults aged 18 years or older; had medical, surgical, or trauma admitting diagnoses; and received mechanical ventilation predicted to last at least 72 h from the time of randomization. Patients were excluded if, at the time of eligibility screening, they had received mechanical ventilation for greater than 72 h; were immunocompromised and at increased risk of iatrogenic probiotic infection or endovascular infection; had primary severe acute pancreatitis; had percutaneous feeding tubes; were unable to receive enteral medications; or if withdrawal of advanced life support was planned. Patients enrolled in a potentially confounding trial (i.e., the Cellular Immunotherapy for Septic Shock [CISS] Observational Study [25], IRC002 and IRC005 Trials [26, 27], Clinical Randomisation of an Antifibrinolytic in Significant Head injury [CRASH-3] Trial [28], Selective Decontamination of the Digestive Tract in Intensive Care Unit [SUDDICU] Trial [29], and Nimodipine Microparticles to Enhance Recovery While Reducing Toxicity After Subarachnoid Hemorrhage [NEWTON] Trial [30]) were also excluded.

Patients were randomly allocated to receive either probiotics (1 × 10^10^ colony forming units of *L. rhamnosus* GG) or an enteral placebo using a 1:1 ratio, stratified by center and admission diagnostic category (i.e., medical, surgical, trauma). Patients, next of kin, and all clinical and research staff were blinded to the allocation [14]. The allocated intervention was administered twice daily for up to 60 days, until ICU discharge, or until an adverse event occurred, which was defined as *Lactobacillus* species isolation from a sterile site or cultured as the sole or predominant organism from a nonsterile site [14]. Both before and during ICU admission, the study team tracked patient coenrollment data, including the number of coenrollment events and name of the coenrolled studies.

The primary outcome of PROSPECT was the incidence of VAP, defined as the presence of new, progressive, or persistent infiltrate on chest radiograph after at least 2 days of mechanical ventilation, plus at least 2 of the following criteria: a fever above 38˚C; white blood cell count less than 3 × 10^6^/L or exceeding 10 × 10^6^/L; or purulent sputum [14].

### Data analysis

We present the coenrollment-related characteristics of patients by treatment group using descriptive statistics, reporting the number of coenrolled patients, number of patients coenrolled into a randomized trial, the number of coenrollment events, and the number of studies into which patients were coenrolled. We also detail the characteristics of coenrolled studies, including the number of patients coenrolled, informed consent model, study design, study affiliation, number of coenrollment events involving studies affiliated with the Canadian Critical Care Trials Group (CCCTG), and funding sources. We present the data in tables, reporting continuous variables as means with standard deviations (SDs) and categorical variables as counts with percentages.

To assess the sensitivity of the treatment effect of probiotics to coenrollment on the primary outcome of VAP, we performed a Cox proportional hazards analysis, stratified by center and admission diagnosis and including independent variables of treatment allocation, coenrollment status, and interaction between treatment and coenrollment status. We report the incidence of VAP in each treatment group stratified by coenrollment status using hazard ratios (HRs) along with corresponding 95% CIs for the two subgroups of coenrolled and non-coenrolled patients and the interaction *p*-value.

For the secondary objective of exploring differences in baseline patient characteristics between coenrolled and non-coenrolled patients, we report descriptive statistics for the following patient-level variables: age; sex; Acute Physiology and Chronic Health Evaluation (APACHE) II score [31]; Clinical Frailty Scale [32]; inotrope or vasopressor infusion use; dialysis use; and individual granting informed consent (i.e., substitute decision-maker vs. patient). Similarly, to explore differences in center-level characteristics between coenrolling and non-coenrolling centers, we report descriptive statistics for the following center-level variables: number of ICU beds; total years of PROSPECT participation; year of PROSPECT site initiation; years of site investigator and lead research coordinator trial experience; hospital type (community or academic); and country. We present the results in tables, reporting continuous variables as means (SDs) and categorical variables as counts with percentages.

To identify factors associated with coenrollment, we performed multilevel logistic regression, with a random intercept to account for clustering at the center-level. Both center-level (i.e., number of ICU beds; total years of PROSPECT participation; site investigator experience; lead research coordinator experience; hospital type) and patient-level (i.e., age, sex, APACHE II score; person providing informed consent) characteristics were included as independent variables. We did not include Clinical Frailty Score in the model as this was not collected in the early phase of the trial, and thus ~ 18% of patients had missing data for this variable. We report the odds ratios (ORs) with corresponding 95% CIs for each independent variable (ORs for categorical variables were presented for n-1 categories, with a reference group).

Finally, to explore the relationship between coenrollment and adverse events, we performed Fisher’s exact tests comparing coenrolled and non-coenrolled patients, reporting as counts (with relative percentages) and the associated *p*-value. Adverse events were defined as the isolation of *Lactobacillus* species in a culture from a sterile site or as the sole or predominant organism cultured from a nonsterile site, as above. Serious adverse events were those *Lactobacillus* isolates resulting in persistent or significant disability or incapacity or were life-threatening or resulting in death. We also conducted post hoc Fisher’s exact tests comparing the rate of adverse events between coenrolled and non-coenrolled within each treatment group, reporting the *p*-value.

As study drug interaction is most conceptually plausible in patients coenrolled into another randomized trial, we performed several post hoc tests exploring the sensitivity of treatment effects to coenrollment in another randomized trial (vs. coenrollment in an observational study or no coenrollment). Studies considered as randomized trials included parallel-group, cluster, and adaptive platform randomized trials. For the primary outcome of VAP, we performed a Cox proportional hazards analysis similar to our primary analysis, but including as independent variables treatment allocation, coenrollment in a randomized trial, and interaction between treatment and coenrollment in a randomized trial. We also performed post hoc Fisher’s exact tests to explore the relationship between adverse event rates and coenrollment in a randomized trial; this was done for the overall sample and within individual treatment groups.

Lastly, given the association between disease severity and adverse events [33], we performed a post hoc Student’s *t*-test comparing APACHE II scores between patients who experienced an adverse event and those who did not. We describe the results in tables, reporting the mean scores for each group and associated *p*-value.

All analyses were conducted by the lead author [AT] using SPSS 28.0 and blindly replicated by the trial analyst [DA]. A *p*-value less than 0.05 was considered statistically significant [34].

## Results

This study included all 2650 patients from the PROSPECT trial, 1318 of whom were randomized to the probiotics group and 1332 to placebo. The trial participants had a mean age of 59.8 years (SD 16.5) and 40.1% were female. Additional details are reported in the original publication [14].

### Patient-level coenrollment characteristics

A total of 568 patients (21.4%) were coenrolled in another study: 298 (22.6%) in the probiotics group and 270 (20.3%) in the placebo group. Of the 568 patients who were coenrolled, 328 (12.4%) were coenrolled into at least one RCT. The total number of coenrollment events was 680, as 97 (3.7%) patients were coenrolled in two or more additional studies. A full description of patient coenrollment characteristics is presented in Table 1.

**Table 1:** Co-enrolled patient characteristics

Patient-level characteristics	*L. rhamnosus* GG *n* = 1318	Placebo *n* = 1332	Total *n* = 2650
**Coenrolled patients**	298 (22.6%)	270 (20.3%)	568 (21.4%)
**Coenrolled into an RCT**	167 (12.7%)	161 (12.1%)	328 (12.4%)
**Coenrollment events (Number of times a patient was included in another study)**	359	321	680
**Number of other studies in which patients were coenrolled**			
0 1 2 3 4	1020 (77.4%) 244 (18.5%) 49 (3.7%) 3 (0.2%) 2 (0.2%)	1062 (79.7%) 227 (17.0%) 36 (2.7%) 6 (0.5%) 1 (0.1%)	2082 (78.6%) 471 (17.8%) 85 (3.2%) 9 (0.3%) 3 (0.1%)

*L. rhamnosus GG, Lactobacillus rhamnosus* GG, *RCT* Randomized controlled trial

### Study-level coenrollment characteristics

The 568 coenrolled patients were coenrolled into 115 different studies across centers. The majority of these studies used an a priori informed consent model (102, 88.7%) and were academically funded (107, 93.0%). With respect to the design of coenrolled studies, 72 (62.6%) were observational studies and 43 (37.4%) were randomized trials, two of which were cluster randomized trials and one of which used a factorial design. Some (30; 26.1%) coenrolled studies were affiliated with the CCCTG, which accounted for 270 out of 680 coenrollment events. Further description of the characteristics of studies in which patients were coenrolled is shown in Table 2 (by coenrollment event in Supplementary Table 2).

**Table 2:** Coenrolled study characteristics

Study-level characteristics	*n* = 115 coenrolled studies
**Main informed consent model for the coenrolled studies**	
A priori informed consent Consent to continue / deferred consent Waived consent	102 (88.7%) 4 (3.5%) 9 (7.8%)
**Design**	
Observational Randomized controlled trial Cluster randomized controlled trial Factorial randomized trial	72 (62.6%) 40 (34.8%) 2 (1.7%) 1 (0.9%)
**Study affiliation with Canadian Critical Care Trials Group**	
Yes No	30 (26.1%) 85 (73.9%)
**Study funding**	
Academic Industry Academic/industry partnership Local	107 (93.0%) 5 (4.3%) 2 (1.7%) 1 (0.9%)

The study with the highest proportion of coenrollment events was the FAST Trial (39 coenrollment events), a factorial randomized trial which compared spontaneous breathing trial (SBT) techniques (i.e., T-piece; Pressure Support) and frequency (once vs twice daily) [35]. Other studies with a high proportion of coenrollment events included the diarrhea: interventions, consequences and epidemiology in the intensive care unit (DICE-ICU) observational study [36], the end-of-life 3 Wishes Project [37], a randomized trial testing adjunctive intermittent pneumatic compression for preventing venous thromboembolism (i.e., the PREVENT trial) [38], and the Standard vs. Accelerated Initiation of Renal Replacement Therapy (STARRT-AKI) randomized trial [39]. The complete list of coenrolled studies is presented in Supplementary Table 3.

### Sensitivity of probiotic treatment effects to coenrollment

We found coenrollment in any study had no influence on the treatment effect of probiotics in the primary outcome of VAP (*p* = 0.630). In both coenrolled and non-coenrolled patients, probiotics conferred no benefit in terms of preventing VAP (Table 3). Similarly, our post hoc analysis found coenrollment in an RCT to have no influence on the treatment effect in the primary outcome of VAP (*p* = 0.967) (Supplementary Table 4).

**Table 3:** Effect of coenrollment on probiotic treatment effect on the primary outcome of ventilator-associated pneumonia

Coenrollment status^1^	Probiotic	Placebo	Hazard ratio (95% CI)	Interaction *P* value^2^
Yes	67/298 (22.5%)	54/270 (20.0%)	1.12 (0.77, 1.63)	0.630
No	222/1020 (21.8%)	230/1062 (21.7%)	1.01 (0.83, 1.22)	

^1^Coenrolled in another study prior to, concurrent with, or after PROSPECT enrollment

^2^Cox proportional hazards model (adjusted for center and admission diagnosis) with independent variables of treatment and coenrollment status plus interaction between treatment and coenrollment status

### Differences between coenrolled and non-coenrolled patients and centers

Coenrolled patients had a higher average disease severity, a higher degree of frailty, a higher proportion of dialysis use, and a lower rate of consent by a substitute decision-maker. However, age and sex were similar in coenrolled and non-coenrolled patients (Table 4).

**Table 4:** Baseline patient characteristics between coenrolled and non-coenrolled patients

Baseline characteristics (*n* = 2650)	Coenrolled patients *n* = 568	Non-coenrolled patients *n* = 2082
**Age** in years, mean (SD)	60.3 (16.4)	59.7 (16.5)
**Female**, *n* (%)	229 (40.3%)	834 (40.1%)
**APACHE II score**, mean (SD)	23.0 (7.5)	21.8 (7.9)
**Clinical Frailty Scale ≥ 5** (*n* = 2182), *n* (%)	115/444 (25.9%)	357/1,738 (20.5%)
**Inotropes or vasopressors**, *n* (%)	338 (59.5%)	1283 (61.6%)
**Dialysis,** *n* (%)	62 (10.9%)	153 (7.3%)
**Individual granting informed consent for PROSPECT** (*n* = 2641), *n* (%)		
Substitute decision-maker Patient	537/566 (94.9%) 29/566 (5.1%)	2023/2,075 (97.5%) 52/2,075 (2.5%)

*APACHE* Acute Physiology And Chronic Health Evaluation

Centers that coenrolled patients were of larger size, with a longer duration of participation in PROSPECT. There was also a greater proportion of academic hospitals and Canadian centers among coenrolling centers compared to non-coenrolling centers (Table 5).

**Table 5:** Center-level characteristics between coenrolling and non-coenrolling centers

	**Coenrolling centers** ***n* = 32**	**Non-coenrolling centers** ***n* = 12**
**Number of ICU beds screened for PROSPECT**		
< 15 15–20 > 20 beds	2 (6.3%) 9 (28.1%) 21 (65.6%)	1 (8.3%) 5 (41.7%) 6 (50.0%)
**Total years of PROSPECT participation**, mean (SD)	3.02 (1.48)	0.97 (0.58)
**Year when PROSPECT initiated**		
1 (Oct 2013–Sept 2014) 2 (Oct 2014–Sept 2015) 3 (Oct 2015–Sept 2016) 4 (Oct 2016–Sept 2017) 5 (Oct 2017–Sept 2018)	11 (34.4%) 2 (6.3%) 9 (28.1%) 6 (18.8%) 4 (12.5%)	1 (8.3%) 1 (8.3%) 2 (16.7%) 4 (33.3%) 4 (33.3%)
**Site investigator trial experience**		
Early career (0–5 years of research) Mid-career (6–15 years) Senior career (> 15 years)	5 (15.6%) 15 (46.9%) 12 (37.5%)	3 (25.0%) 5 (41.7%) 4 (33.3%)
**Lead research coordinator trial experience**		
Early career (0–5 years of experience) Mid-career (6–15 years) Senior career (> 15 years)	7 (21.9%) 16 (50.0%) 9 (28.1%)	5 (41.7%) 2 (16.7%) 5 (41.7%)
**Hospital type**		
Community Academic	4 (12.5%) 28 (87.5%)	3 (25.0%) 9 (75.0%)
**Location**		
Canada United States Saudi Arabia	31 (96.9%) 0 (0.0%) 1 (3.1)	10 (83.3%) 2 (16.7%) 0 (0.0%)

*ICU* Intensive care unit

### Patient- and center-level factors associated with coenrollment

Multilevel logistic regression found coenrollment to be associated with longer duration of recruitment in PROSPECT (*p* = 0.047) and greater patient disease severity (*p* = 0.038) (Table 6). No other patient- or center-level factors were associated with coenrollment.

**Table 6:** Patient- and center-level factors associated with coenrollment

Independent variables (*n* = 2641)	OR (95% CI)	*P*-value
**Center level**		
**Number of ICU beds screened for PROSPECT**		
< 15	Ref	
15–20	0.49 (0.59, 4.07)	0.509
> 20 beds	0.34 (0.04, 2.75)	0.308
**Total years of PROSPECT participation (per 1-year increase)**	1.42 (1.01, 1.99)	0.047
**Site investigator trial experience**		
Early career (0–5 years of research)	Ref	
Mid-career (6–15 years)	2.03 (0.20, 20.73)	0.552
Senior career (> 15 years)	2.82 (0.26, 31.02)	0.396
**Lead research coordinator trial experience**		
Early career (0–5 years of research experience)	Ref	
Mid-career (6–15 years)	2.00 (0.40, 10.08)	0.400
Senior career (> 15 years)	1.47 (0.26, 8.43)	0.664
**Hospital type**		
Community	Ref	
Academic	1.20 (0.13, 10.90)	0.872
**Patient level**		
** Age (years; per 10-year increase)**	0.98 (0.91, 1.04)	0.465
**Sex**		
Male	Ref	
Female	0.98 (0.79, 1.21)	0.846
**APACHE II score (per 5-unit increase)**	1.08 (1.00, 1.16)	0.038
**Informed consent grantor**		
Substitute decision-maker	Ref	
Patient	1.60 (0.96, 2.66)	0.073

*ICU*, intensive care unit; *OR*, odds ratio; *APACHE*, Acute Physiology and Chronic Health Evaluation

### Adverse events

In total, 14 adverse events and 2 serious adverse events, all of which were isolation of *Lactobacillus* species, were reported in the PROSPECT trial. Of these, 15 were in the probiotics group and 1 in the placebo group [14]. The isolates originated from various sites: blood [10]; urine [2]; blood and hepatic abscess [1]; intra-abdominal abscess [1]; peritoneal fluid [1]; and pleural fluid [1].

Overall, 8/568 (1.4%) coenrolled patients (9 coenrollment events in 8 different studies) experienced a probiotic-associated *Lactobacillus* spp. isolation, compared to 8/2082 (0.4%) non-coenrolled patients (*p* = 0.011); this low overall rate was expected given how rare it is to identify *Lactobacillus* spp. isolates in practice.

We conducted exploratory post hoc testing by treatment group, finding a significantly higher rate of adverse events in coenrolled patients randomized to probiotics (8/298; 2.7%) compared to non-coenrolled patients randomized to probiotics (7/1020; 0.7%) (*p* = 0.009)) (Table 7). In the placebo group, no difference in the incidence of adverse events was observed between coenrolled and non-coenrolled patients (*p* = 1.000).

**Table 7:** Adverse event rates according to coenrollment status

Group	Coenrollment status	Number of adverse events (*n* = 16)	*P*-value
Overall	Coenrolled (*n* = 568)	8 (1.4%)	0.011
	Not coenrolled (*n* = 2082)	8 (0.4%)	
Probiotic	Coenrolled (*n* = 298)	8 (2.7%)	0.009^1^
	Not coenrolled (*n* = 1020)	7 (0.7%)	
Placebo	Coenrolled (*n* = 270)	0 (0.0%)	1.000^1^
	Not coenrolled (*n* = 1062)	1 (0.1%)	

^1^Post hoc Fisher’s exact test

We also conducted further post hoc exploratory analyses comparing the rate of adverse events between patients who were coenrolled in an RCT compared to those coenrolled in an observational study or no other study. In these analyses, we found no difference in the rate of adverse events in the overall sample (*p* = 0.126), in the probiotics group (*p* = 0.111), or the placebo group (*p* = 1.000) (Supplementary Table 5).

Given the relationship between disease severity and adverse events [33] and the results of our regression analyses indicating an association between coenrollment and disease severity, we conducted a final post hoc exploratory analysis comparing APACHE II scores between patients with adverse events and patients without adverse events to explore whether the difference in adverse events between coenrolled and non-coenrolled patients was confounded by a difference in disease severity. We found no difference in APACHE II scores between patients with and without adverse events (*p* = 0.856) (Supplementary Table 6).

## Discussion

In this international, blinded trial evaluating the effect of probiotics on VAP during critical illness [14], 21.4% of patients were coenrolled into at least one other study. We found no impact of coenrollment on the effect of probiotics in the development of VAP. Coenrolled PROSPECT patients were more likely to have higher disease severity and to be recruited from a center which had longer duration of recruitment in PROSPECT. Adverse events were more common in coenrolled patients compared to non-coenrolled patients. However, notably, post hoc analyses focusing on coenrollment in only an RCT which was associated with exposure to other interventions (vs. no coenrollment or coenrollment in an observational study) found no differences in the rate of adverse events.

Our findings align with the original conclusions of the PROSPECT trial that probiotics are not beneficial for the prevention of VAP [14]. A substantial proportion of the previous randomized trials reporting efficacy were of high risk of bias, which are susceptible to exaggerated treatment effects [40]; removing these studies eliminates the effect of probiotics on VAP [15, 16]. Studies examining probiotic use in mechanically ventilated, critically ill patients are thus an example of how treatment estimates can meaningfully differ based on study design.

We found that coenrolled patients, compared to non-coenrolled patients, were more likely to have an adverse event in the form of an isolation of the probiotic bacteria *Lactobacillus rhamnosus* GG. This was particularly evident in the probiotics group, where a post hoc analysis found a fourfold increase in probiotic isolates among coenrolled patients compared to non-coenrolled patients. Whether isolates reflected true infection or post-sample collection contamination from the hands of healthcare workers was not clear in all cases [14]. Further, whether these isolates are a consequence of more intense observation (e.g., more frequent blood cultures drawn) and thus ascertainment bias is uncertain. We examined whether coenrollment was associated with high disease severity, which could also be associated with a higher likelihood of adverse events [33]. However, post hoc testing found no relationship between adverse events and disease severity. Exposure to multiple study treatments [41, 42] as occurs with coenrollment may also be associated with an increased risk of adverse events. However, one of our a priori criteria for selecting eligible studies for coenrollment was the lack of biological plausibility for an interaction between probiotics in PROSPECT and other studies—trials in which the intervention was very unlikely to interact with probiotics, as well as observational studies with no intervention (representing 62.6% of coenrolled studies). Importantly, we did not detect any differences in adverse event rate between patients coenrolled in an interventional RCT vs. patients not coenrolled in an RCT in our additional post hoc testing. Given the small number of adverse events and large proportion of observational studies among the coenrolled studies, the initial findings may have been due to chance. Regardless, coenrolled patients should be diligently monitored to mitigate any potential health risks, and probiotics trials should continue to document probiotic-associated bacterial isolates.

We found in multivariable analyses that coenrolled patients were more likely to have a greater disease severity (APACHE II score) and to be coenrolled by a center with a longer duration of PROSPECT recruitment. These results are not unexpected in that an association between disease severity and coenrollment status was also observed in the Prophylaxis for Thromboembolism in Critical Care Trial (PROTECT) [20]. The greater likelihood of coenrollment in centers with a longer duration of PROSPECT recruitment may reflect how such centers generally facilitate research. This was illustrated in the Oscillation for ARDS Treated Early Trial (OSCILLATE), where coenrolling centers participated in more RCTs and had a longer duration of OSCILLATE participation compared to non-coenrolling centers [18].

The rate of coenrollment in PROSPECT (21.4%) was similar to that of other ICU trials such as OSCILLATE (23.2%) [18] and PROTECT (19.0%) [20], and our findings that coenrollment did not influence the primary trial treatment effect are consistent with OSCILLATE and PROTECT Trial analyses [18, 20]. However, our results diverge with respect to factors associated with coenrollment. While we found an association between disease severity and coenrollment, reflecting the eligibility criteria of other studies focused on seriously ill patients, we found no association between site investigator or research coordinator trial experience and coenrollment in multivariable analyses, whereas the OSCILLATE Trial found both to be associated with a higher likelihood of coenrollment [18]. Also, we found no association between coenrollment and the individual granting consent in PROSPECT, although in PROTECT, substitute decision-makers were associated with an increased likelihood of coenrollment [20]. These discrepancies further illustrate the complexity of coenrollment and the need to assess the effects of coenrollment within each trial.

We suggest future research evaluating the patterns and consequences of coenrollment in critical care studies. The typical concerns regarding coenrollment in critical care settings—patient safety, internal validity of coenrolled studies, and feasibility—continue to be important topics of consideration and debate. While coenrollment generates research efficiency gains and provides opportunities for patients to participate in more research and potentially receive superior treatment (although the opposite is possible) [43, 44], it is important to assess possible interactions between study interventions prior to coenrollment, to thoughtfully evaluate the possible consent burden and stress on patients, their families, and health care teams, and to consider potential logistical and organizational challenges, ensuring that coenrollment does more good than harm. Current research has found that patients and caregivers are willing to participate in more than one study [45–47], which suggests that coenrollment is manageable in several settings. However, continued exploration of these contextual factors is needed. Our findings also raise important questions regarding the conduct of coenrolling in platform trials: future analyses should determine whether coenrollment modifies effect estimates for patients enrolled in more than one domain of a platform trial, particularly if adaptive randomization results in differential recruitment to the control arm, if interventions are unblinded, and if cointerventions are unequally distributed.

This study has several strengths. First, the large, international sample and detailed collection of coenrollment data not typically captured in trials allowed analyses exploring the patterns, predictors, and effects of coenrollment. Second, we studied a critically ill population at high risk of morbidity and mortality, focusing on research design and implementation to improve patient outcomes and the quality of care [48]. Third, we were guided by pre-specified hypotheses (Supplementary Table 1) and an a priori protocol and statistical analysis plan [34], which increases the transparency and validity of our findings. Fourth, we performed a multilevel logistic regression analysis to explore factors associated with coenrollment, including a random intercept to account for clustering at the center-level. This improves validity by generating a more accurate estimate of relationships in clustered data [49]. Fifth, we performed all analyses independently and in duplicate, minimizing the risk of human to ensure the validity of our findings. These results could help to inform practices and policies pertaining to coenrollment within and beyond the field of critical care.

This study has some limitations. First, we have no information on the overall number of studies ongoing at each center, which would help to contextualize findings. Second, we lack data on potential studies that were declined for coenrollment and the reasons for declining by patients and surrogate decision-makers, limiting our ability to identify patterns in the consent encounters and decisions made. Third, within coenrolled RCTs, we did not distinguish those testing novel interventions (of which there were few) from those testing common interventions in practice. Fourth, if probiotics had reduced VAP in this trial, it is unknown whether coenrollment would have modified those findings. Fifth, this study did not benefit from qualitative data elicited from patients, family members, clinicians, research coordinators, investigators, research ethics boards, or other stakeholders; however, clinicians, research coordinators, and investigators planned this study, interpreted the analyses, and wrote this report. Lastly, we did not stratify our analyses by coenrollment timing as such data was not uniformly available across all patients.

## Conclusion

Coenrollment occurred in one-fifth of patients enrolled in this international, blinded probiotics trial. Coenrollment did not influence the treatment effect of probiotics in the primary outcome of VAP.

## Supplementary Information

Additional file 1: Supplementary Tables 1–6.

## Data Availability

The datasets generated and/or analyzed during the current study are not publicly available as trial participants did not grant permission for that during the informed consent process.

## Abbreviations

PROSPECT: Probiotics: Prevention of Severe Pneumonia and Endotracheal Colonization Trial
RCT: Randomized controlled trial
*L. **rhamnosus** GG*: *Lactobacillus **rhamnosus* GG
VAP: Ventilator-associated pneumonia
ICU: Intensive care unit
CISS: Cellular Immunotherapy for Septic Shock
SUDDICU: Selective Decontamination of the Digestive Tract in Intensive Care Unit
NEWTON: Nimodipine Microparticles to Enhance Recovery While Reducing Toxicity After Subarachnoid Hemorrhage
SD: Standard deviation
CI: Confidence intervals
APACHE: Acute Physiology and Chronic Health Evaluation
OR: Odds ratio
CCCTG: Canadian Critical Care Trials Group
PROTECT: Prophylaxis for ThromboEmbolism in Critical Care Trial
OSCILLATE: Oscillation for ARDS Treated Early Trial
