# Supplementary material for: Coenrollment of critically ill patients in PROSPECT: characteristics and association with treatment efficacy and safety
Source: Trials. 2025 Sep 26;26:370. doi: 10.1186/s13063-025-09028-w (PMC12465574; doi:10.1186/s13063-025-09028-w)
Supplement: Supplementary file 1 — Additional file 1: Supplementary Tables 1–6. [file 13063_2025_9028_MOESM1_ESM.docx]

**Supplementary Table 1: Results of Hypothesis Testing**

| **Objectives** | **Hypothesis** | **Results of Hypothesis Testing** |
| --- | --- | --- |
| **Primary** | | |
| 1. Evaluate the sensitivity of the effects of probiotics to patient coenrollment in the primary outcome | 1. Co-enrollment will not modify the treatment effect of probiotics on the outcome of VAP | 1) Confirmed |
| **Secondary** | | |
| 1. Describe characteristics of coenrolled patients | N/A | N/A |
| 1. Describe characteristics of coenrolled studies | N/A | N/A |
| 1. Explore differences between coenrolled and non-coenrolled patients | N/A | N/A |
| 1. Explore differences between coenrolling and non-coenrolling centers | N/A | N/A |
| 1. Identify factors associated with coenrollment | Coenrollment will be associated with:   1. Less seriously ill patients (APACHE II) 2. Consent granted by an SDM 3. Academic centers 4. Larger center size (more ICU beds) 5. Experienced site investigators and lead research coordinators | 1) Not confirmed  2) Not confirmed  3) Not confirmed  4) Not confirmed  5) Not confirmed |
| 1. Explore relationship between coenrollment and adverse events | 1. Coenrollment will not modify the rate of adverse events in both the probiotics and control group | 1) Not confirmed |
| *VAP = ventilator-associated pneumonia; SDM = substitute decision-maker* | | |

**Supplementary Table 2: Coenrollment Study Characteristics**

| **Study Level Characteristic** | **n=680 Coenrollment Events** |
| --- | --- |
| **Informed Consent Model for Coenrolled Study**  A priori  Deferred  Waived consent | 574 (84.4)  19 (2.8)  87 (12.8) |
| **Design of Coenrolled Study**  RCT  Cluster RCT  Prospective Observational Study | 319 (46.9)  30 (4.4)  331 (48.7) |
| **Coenrolled Study Affiliated with Canadian Critical Care Trials Group**  Yes  No | 270 (39.7)  410 (60.3) |
| **Funding of Coenrolled Study**  Academic  Academic and Industry  Industry  Local | 624 (91.8)  9 (1.3)  43 (6.3)  4 (0.6) |


**Supplementary Table 3: Coenrolled Studies**

| **Supplementary Table 1: Coenrolled Studies** | ***L. rhamnosus GG***  **n=359 coenrollment events** | **Placebo**  **n=321 coenrollment events** | **Total**  **n=680 coenrollment events** |
| --- | --- | --- | --- |
| FAST | 22 (6.1%) | 17 (5.3%) | 39 (5.7%) |
| DICE | 18 (5.0%) | 19 (5.9%) | 37 (5.4%) |
| 3 Wishes Demonstration Project | 21 (5.8%) | 16 (5.0%) | 37 (5.4%) |
| PREVENT | 18 (5.0%) | 18 (5.6%) | 36 (5.3%) |
| STARRT-AKI | 24 (6.7%) | 10 (3.1%) | 34 (5.0%) |
| CYCLE | 12 (3.3%) | 16 (5.0%) | 28 (4.1%) |
| Propel | 10 (2.8%) | 16 (5.0%) | 26 (3.8%) |
| BALANCE | 17 (4.7%) | 8 (2.5%) | 25 (3.7%) |
| STRATUS | 11 (3.1%) | 11 (3.4%) | 22 (3.2%) |
| BIA | 10 (2.8%) | 9 (2.9%) | 19 (2.8%) |
| Extubation Advisor | 7 (1.9%) | 8 (2.5%) | 15 (2.2%) |
| Replenish | 7 (1.9%) | 7 (2.2%) | 14 (2.1%) |
| Confocal | 8 (2.2%) | 4 (1.2%) | 12 (1.8%) |
| FORECAST | 8 (2.2%) | 4 (1.2%) | 12 (1.8%) |
| ARDS (Oscillate) | 6 (1.7%) | 4 (1.2%) | 10 (1.5%) |
| DDePART | 4 (1.1%) | 6 (1.9%) | 10 (1.5%) |
| IMPACT | 2 (0.6%) | 8 (2.5%) | 10 (1.5%) |
| DONATE | 4 (1.1%) | 5 (1.6%) | 9 (1.3%) |
| MIME | 5 (1.4%) | 4 (1.2%) | 9 (1.3%) |
| TryCYCLE | 3 (0.8%) | 5 (1.6%) | 8 (1.2%) |
| INSIST | 4 (1.1%) | 4 (1.2%) | 8 (1.2%) |
| PROMIZING | 3 (0.8%) | 5 (1.6%) | 8 (1.2%) |
| Sahara | 1 (0.3%) | 7 (2.2%) | 8 (1.2%) |
| FLUID | 2 (0.6%) | 6 (1.9%) | 8 (1.2%) |
| ABLE | 4 (1.1%) | 3 (0.9%) | 7 (1.0%) |
| REVISE | 3 (0.8%) | 4 (1.2%) | 7 (1.0%) |
| OPTIMAL-AKI | 4 (1.1%) | 2 (0.6%) | 6 (0.9%) |
| EPVENT2 | 3 (0.8%) | 3 (0.9%) | 6 (0.9%) |
| TOPPIT | 2 (0.6%) | 4 (1.2%) | 6 (0.9%) |
| Prognosis TBI study | 3 (0.8%) | 2 (0.6%) | 5 (0.7%) |
| PERMIT | 4 (1.1%) | 1 (0.3%) | 5 (0.7%) |
| Enoxaparin Xa Levels in RF | 3 (0.8%) | 2 (0.6%) | 5 (0.7%) |
| PRO-TROPICS | 2 (0.6%) | 3 (0.9%) | 5 (0.7%) |
| DIVIP | 2 (0.6%) | 3 (0.9%) | 5 (0.7%) |
| CPOT | 3 (0.8%) | 2 (0.6%) | 5 (0.7%) |
| COMIC | 4 (1.1%) | 1 (0.3%) | 5 (0.7%) |
| RUBIC | 2 (0.6%) | 3 (0.9%) | 5 (0.7%) |
| HEMOTION | 1 (0.3%) | 4 (1.2%) | 5 (0.7%) |
| CHG-Lock | 2 (0.6%) | 3 (0.9%) | 5 (0.7%) |
| CONFOCAL 2 | 4 (1.1%) | 1 (0.3%) | 5 (0.7%) |
| EFFORT | 3 (0.8%) | 2 (0.6%) | 5 (0.7%) |
| SARI | 2 (0.6%) | 2 (0.6%) | 4 (0.6%) |
| AFOTS | 2 (0.6%) | 2 (0.6%) | 4 (0.6%) |
| ECHO-AKI | 3 (0.8%) | 1 (0.3%) | 4 (0.6%) |
| SLEE-WE | 3 (0.8%) | 1 (0.3%) | 4 (0.6%) |
| Preventing PTSD in ICU survivors | 2 (0.6%) | 2 (0.6%) | 4 (0.6%) |
| STORM | 2 (0.6%) | 2 (0.6%) | 4 (0.6%) |
| Optical Device | 1 (0.3%) | 3 (0.9%) | 4 (0.6%) |
| HALO | 2 (0.6%) | 1 (0.3%) | 3 (0.4%) |
| Nutriate | 2 (0.6%) | 1 (0.3%) | 3 (0.4%) |
| OVATION Observational | 3 (0.8%) | 0 (0.0%) | 3 (0.4%) |
| APRV/BIPAP with spontaneous breathing | 2 (0.6%) | 1 (0.3%) | 3 (0.4%) |
| FLAIL | 2 (0.6%) | 1 (0.3%) | 3 (0.4%) |
| FADE | 0 (0.0%) | 3 (0.9%) | 3 (0.4%) |
| DIAPHRAGM | 3 (0.8%) | 0 (0.0%) | 3 (0.4%) |
| RELEASE/SENIOR | 1 (0.3%) | 2 (0.6%) | 3 (0.4%) |
| COGWELL | 2 (0.6%) | 1 (0.3%) | 3 (0.4%) |
| Derecruitment | 2 (0.6%) | 1 (0.3%) | 3 (0.4%) |
| Frailty Study | 1 (0.3%) | 2 (0.6%) | 3 (0.4%) |
| NEURO-ETT | 2 (0.6%) | 1 (0.3%) | 3 (0.4%) |
| PREDICT | 1 (0.3%) | 2 (0.6%) | 3 (0.4%) |
| SPICEIII | 2 (0.6%) | 1 (0.3%) | 3 (0.4%) |
| Flowly | 1 (0.3%) | 2 (0.6%) | 3 (0.4%) |
| OVATION | 1 (0.3%) | 1 (0.3%) | 2 (0.3%) |
| IOSwean | 2 (0.6%) | 0 (0.0%) | 2 (0.3%) |
| Eadi to optimize ventilation | 1 (0.3%) | 1 (0.3%) | 2 (0.3%) |
| Horner's Syndrome CV Cath | 1 (0.3%) | 1 (0.3%) | 2 (0.3%) |
| Community Acquired Pneumonia | 2 (0.6%) | 0 (0.0%) | 2 (0.3%) |
| GIFT | 0 (0.0%) | 2 (0.6%) | 2 (0.3%) |
| OWED | 1 (0.3%) | 1 (0.3%) | 2 (0.3%) |
| EFRAIM | 2 (0.6%) | 0 (0.0%) | 2 (0.3%) |
| SOLVE-ARDS Study | 2 (0.6%) | 0 (0.0%) | 2 (0.3%) |
| ISAT 2 | 1 (0.3%) | 1 (0.3%) | 2 (0.3%) |
| SEPSIS | 2 (0.6%) | 0 (0.0%) | 2 (0.3%) |
| Treg Cell | 2 (0.6%) | 0 (0.0%) | 2 (0.3%) |
| COMA | 1 (0.3%) | 1 (0.3%) | 2 (0.3%) |
| OVATION-65 | 1 (0.3%) | 1 (0.3%) | 2 (0.3%) |
| Compare | 1 (0.3%) | 1 (0.3%) | 2 (0.3%) |
| Projet Pupilles | 2 (0.6%) | 0 (0.0%) | 2 (0.3%) |
| INDex | 1 (0.3%) | 1 (0.3%) | 2 (0.3%) |
| FISSH | 1 (0.3%) | 1 (0.3%) | 2 (0.3%) |
| ICUAW | 1 (0.3%) | 1 (0.3%) | 2 (0.3%) |
| MANAGE | 1 (0.3%) | 0 (0.0%) | 1 (0.1%) |
| ABLE Arms | 1 (0.3%) | 0 (0.0%) | 1 (0.1%) |
| VALTS | 1 (0.3%) | 0 (0.0%) | 1 (0.1%) |
| Molecular Signature of Sepsis | 0 (0.0%) | 1 (0.3%) | 1 (0.1%) |
| Sentinel Surveillance | 1 (0.3%) | 0 (0.0%) | 1 (0.1%) |
| Free Cortisol in Sepsis | 1 (0.3%) | 0 (0.0%) | 1 (0.1%) |
| Pathophysiology of Hit | 0 (0.0%) | 1 (0.3%) | 1 (0.1%) |
| DELTA | 1 (0.3%) | 0 (0.0%) | 1 (0.1%) |
| PMS Scan vs Thorax Echo | 0 (0.0%) | 1 (0.3%) | 1 (0.1%) |
| LICOX | 0 (0.0%) | 1 (0.3%) | 1 (0.1%) |
| High dose steroids-dysphagia post spinal fusion | 1 (0.3%) | 0 (0.0%) | 1 (0.1%) |
| PIC | 0 (0.0%) | 1 (0.3%) | 1 (0.1%) |
| STATUS | 1 (0.3%) | 0 (0.0%) | 1 (0.1%) |
| ELIQUIS | 1 (0.3%) | 0 (0.0%) | 1 (0.1%) |
| Gradient pression artérielle radiale-fémorale | 1 (0.3%) | 0 (0.0%) | 1 (0.1%) |
| CONCEPT | 0 (0.0%) | 1 (0.3%) | 1 (0.1%) |
| CAMP HIBI | 0 (0.0%) | 1 (0.3%) | 1 (0.1%) |
| CAN-TBI | 0 (0.0%) | 1 (0.3%) | 1 (0.1%) |
| USEFUL | 0 (0.0%) | 1 (0.3%) | 1 (0.1%) |
| Pure Alliance | 1 (0.3%) | 0 (0.0%) | 1 (0.1%) |
| SPRINT SARI | 0 (0.0%) | 1 (0.3%) | 1 (0.1%) |
| Patient Centered Outcomes Prognostication in ICU | 1 (0.3%) | 0 (0.0%) | 1 (0.1%) |
| DASH study | 1 (0.3%) | 0 (0.0%) | 1 (0.1%) |
| MINT | 1 (0.3%) | 0 (0.0%) | 1 (0.1%) |
| Mellow | 1 (0.3%) | 0 (0.0%) | 1 (0.1%) |
| TCD Fractality | 0 (0.0%) | 1 (0.3%) | 1 (0.1%) |
| Hidden Awareness | 0 (0.0%) | 1 (0.3%) | 1 (0.1%) |
| Beards | 1 (0.3%) | 0 (0.0%) | 1 (0.1%) |
| COHO | 0 (0.0%) | 1 (0.3%) | 1 (0.1%) |
| Body Image | 0 (0.0%) | 1 (0.3%) | 1 (0.1%) |
| VAP DX | 0 (0.0%) | 1 (0.3%) | 1 (0.1%) |
| Covert Consciousness | 0 (0.0%) | 1 (0.3%) | 1 (0.1%) |
| LEUK Sepsis | 1 (0.3%) | 0 (0.0%) | 1 (0.1%) |

**Supplementary Table 4: Post-hoc Analyses of Effect of RCT Coenrollment on Probiotic Treatment Effect on the Primary Outcome of VAP**

| **Coenrolled in an RCT^1^** | ***L. rhamnosus GG*** | **Placebo** | **Hazard Ratio**  **(95% CI)** | **Interaction *P* value^2^** |
| --- | --- | --- | --- | --- |
| Yes | 38/167 (22.8%) | 34/161 (21.1%) | 1.04 (0.63, 1.72) | 0.967 |
| No | 251/1151 (21.8%) | 250/1171 (21.3%) | 1.03 (0.86, 1.23) |  |
| *^1^* *Coenrolled in an RCT or Cluster RCT (vs. Observational Study or no coenrollment) prior to, concurrent with, or after PROSPECT enrollment*  *^2^ Cox proportional hazards model (by center and admission diagnosis) with independent variables of treatment and coenrollment in an RCT plus interaction between treatment and coenrollment in an RCT* | | | | |

**Supplementary Table 5: Post-hoc Analyses of Adverse Event Rates According by RCT Coenrollment Status**

| **Group** | **Coenrolled in an RCT^1^** | **Number of Adverse Events (n=16)** | **P-value^2^** |
| --- | --- | --- | --- |
| Overall | Yes (n=328) | 4 (1.2%) | 0.126 |
|  | No (n=2322) | 12 (0.5%) |  |
| Probiotic | Yes (n=167) | 4 (2.4%) | 0.111 |
|  | No (n=1151) | 11 (1.0%) |  |
| Placebo | Yes (n=161) | 0 (0.0%) | 1.000 |
|  | No (n=1171) | 1 (0.1%) |  |
| *^1^ Coenrolled in an RCT or Cluster RCT (vs. Observational Study or no coenrollment) prior to, concurrent with, or after PROSPECT enrollment*  *^2^ Post-hoc Fisher’s Exact Test* | | | |

**Supplementary Table 6: Post-hoc Analyses Comparing Disease Severity By Adverse Event**

| **Adverse Event Status** | **APACHE II Score  Mean (SD)** | **P-value^1^** |
| --- | --- | --- |
| Yes (n=16) | 22.38 (5.67) | 0.856 |
| No (n=2,634) | 22.02 (7.83) |  |
| *^1^ Student’s t-test* | | |
